# Cost-effectiveness analysis of ceftazidime-avibactam as definitive treatment for treatment of carbapenem-resistant *Klebsiella pneumoniae* bloodstream infection

**DOI:** 10.3389/fpubh.2023.1118307

**Published:** 2023-02-28

**Authors:** Wenqiang Kong, Xueting Yang, Yunfeng Shu, Shiqin Li, Bihui Song, Kun Yang

**Affiliations:** ^1^Department of Pharmacy, Zi Gong First People's Hospital, Zi Gong, China; ^2^Department of Pharmacy, The First People's Hospital of Yunnan Province, The Affiliated Hospital of Kunming University of Science and Technology, Kun Ming, China; ^3^Department of Hematology, Zigong First People's Hospital, Zigong, China

**Keywords:** ceftazidime-avibactam, polymyxin B, cost-effectiveness, carbapenem-resistant *K. pneumoniae*, bloodstream infection

## Abstract

**Background:**

Ceftazidime-avibactam (CAZ-AVI) is a novel antibiotic that has been confirmed in the United States and China for use in patients with carbapenem-resistant *Klebsiella pneumoniae* (CRKP) bloodstream infection (BSI). However, the cost-effectiveness of CAZ-AVI is unknown in China. This study aimed to evaluate the cost-effectiveness of CAZ-AVI compared to polymyxin B (PMB) monotherapy or PMB-based therapy for the treatment of CRKP BSI from the Chinese healthcare perspective.

**Methods:**

A hybrid decision tree and Markov model were constructed for a hypothetical cohort of patients with CRKP BSI. The time horizon of the Markov model was 5 years with an annual discount rate of 5% used in both costs and quality-adjusted life-years (QALYs). The model data was derived from published literature and publicly available database. Regimens with an incremental cost-effectiveness ratio (ICER) lower than the willingness-to-pay (WTP) threshold of $ 11,600 per QALY were considered cost-effective. Deterministic and probabilistic sensitivity analyses were performed to examine the robustness of model analysis.

**Results:**

In the base-analysis, CAZ-AVI provided an additional 60 QALYs and reduced the cost by $ 2,218,300, yielding an ICER of $ −36,730.9/QALY, well below the WTP threshold of $ 11,600 per QALY when compared with PMB-based therapy. CAZ-AVI provided an additional 350 QALYs and increased the cost of $ 208,400, producing an ICER of $ 591.7/QALY that was below the WTP threshold compared to PMB monotherapy. At a $ 11,600/QALY threshold, results were sensitive to the cost of PMB-based strategy, the cost of CAZ-AVI strategy, the probability of cure with CAZ-AVI, and the probability of cure with PMB or PMB-based therapy. CAZ-AVI was an optimal regimen in 76.9% and 80.8% of 10,000 Monte Carlo simulations at $ 11,600/QALY and $ 34,800/QALY, respectively. Meanwhile, CAZ-AVI was cost-effective at the WTP thresholds of all 31 Chinese provinces in 61.4% (Gansu) to 83.1% (Beijing) of simulations.

**Conclusions:**

Ceftazidime-avibactam is expected to be a cost-effective treatment compared with PMB monotherapy or PMB-based therapy for CRKP BSI from the Chinese healthcare perspective.

## Introduction

Antimicrobial resistance is a significant cause of death worldwide, and led to about 1.27 million deaths in 2019 (1). In recent years, carbapenem-resistant *Klebsiella pneumoniae* (CRKP) has dramatically increased and is highly endemic in many countries, limiting the selection of antibiotic therapy with few available treatment options (2). Resistance to carbapenems has resulted in high mortality and significant socioeconomic burden, particularly among vulnerable populations such as those with hematologic malignancies (3–6). A systematic review and meta-analysis showed that the mortality of patients with bloodstream infection (BSI) caused by CRKP was up to 54.3%, significantly higher than those infected with carbapenem-susceptible *K*. *pneumoniae* (7). CRKP has been listed as a critical priority pathogen for research and development of new antibiotics by the World Health Organization (WHO) (8). However, the prevalence of CRKP is alarmingly increasing. Results from the China Antimicrobial Resistance Surveillance Trial (CARST) Program revealed that carbapenem resistance rates in *K. pneumoniae* isolated from blood rose from 3.3 and 1.6% in the 2011–2012 period to 15.0 and 15.4% in the 2019–2020 period, respectively (9). The most common carbapenemase gene is *bla*_KPC − 2_ among *K. pneumoniae* in China (10).

Combined antimicrobial therapies were recommended, and polymyxin-based regimens such as polymyxin B (PMB) in combination with high-dose meropenem or tigecycline were the most common choice in China until ceftazidime-avibactam (CAZ-AVI) was approved for carbapenem-resistant Enterobacterales (CRE) infections in 2019 (11). PMB is administrated in active form and primarily excreted by non-renal mechanisms, rendering it to achieve peak plasma concentrations more rapidly in that it appears optimal for BSI and is associated with a lower risk of acute kidney injury than colistin colistimethate (12, 13). Approximately 95.8% of CRE are susceptible to PMB (10). Thus, PMB is the most frequently administrated in patients with CRE infections in China. CAZ-AVI is a second-generation β-lactam/β-lactamase inhibitor (BL/BLI) with *in vitro* activity against CRE-producing Ambler class A (e.g., KPC), class C (e.g., AmpC), and some class D (e.g., OXA-48) β-lactamases except for metallo-β-lactamases (e.g., NDM-1) (14). CAZ-AVI showed excellent antibacterial activity in vitro against blaKPC-positive *K. pneumoniae* in China (10). Recently, a meta-analysis including 11 retrospective studies demonstrated that CAZ-AVI had a significantly lower 30-day mortality than other regimens for CRE bacteremia and supported the use of CAZ-AVI in CRE bloodstream infections without additional safety concerns (15). The importance of CAZ-AVI in the treatment of CRE infection has been recognized by WHO and included in the 21st WHO model list of essential medicines (16). CAZ-AVI may be a cost-effective alternative when compared with ceftolozane/tazobactam and meropenem for complicated intra-abdominal infections (cIAIs) in Italy (17). Kongnakorn et al. conducted a cost-effectiveness study and revealed that compared with imipenem, CAZ-AVI was expected to be a cost-effective treatment as empirical treatment for complicated urinary tract infections (cUTIs) in Italy (18). However, Han et al. found that CAZ-AVI was not a cost-effective option as an empirical treatment for cUTIs in China when compared with imipenem based on the results of pharmacoeconomic analysis (19). In the US, Simon et al. found that CAZ-AVI was a more cost-effective option than colistin-based regimens for CRE bacteremia and pneumonia (20).

To our knowledge, no cost-effectiveness analysis has yet been performed to determine the health economic value of CAZ-AVI compared with that of PMB or PMB-based regimens in patients with BSI caused by CRKP in China. Economic evaluation of CAZ-AVI is particularly important, helping to guide its use and compete increased drug treatment cost. Thus, the objective of this study was to compare the cost-effectiveness of CAZ-AVI with that of PMB or PMB-based for the treatment of BSI caused by CRKP from the perspective of Chinese healthcare.

## Methods

### Model structure

**Figure 1:**
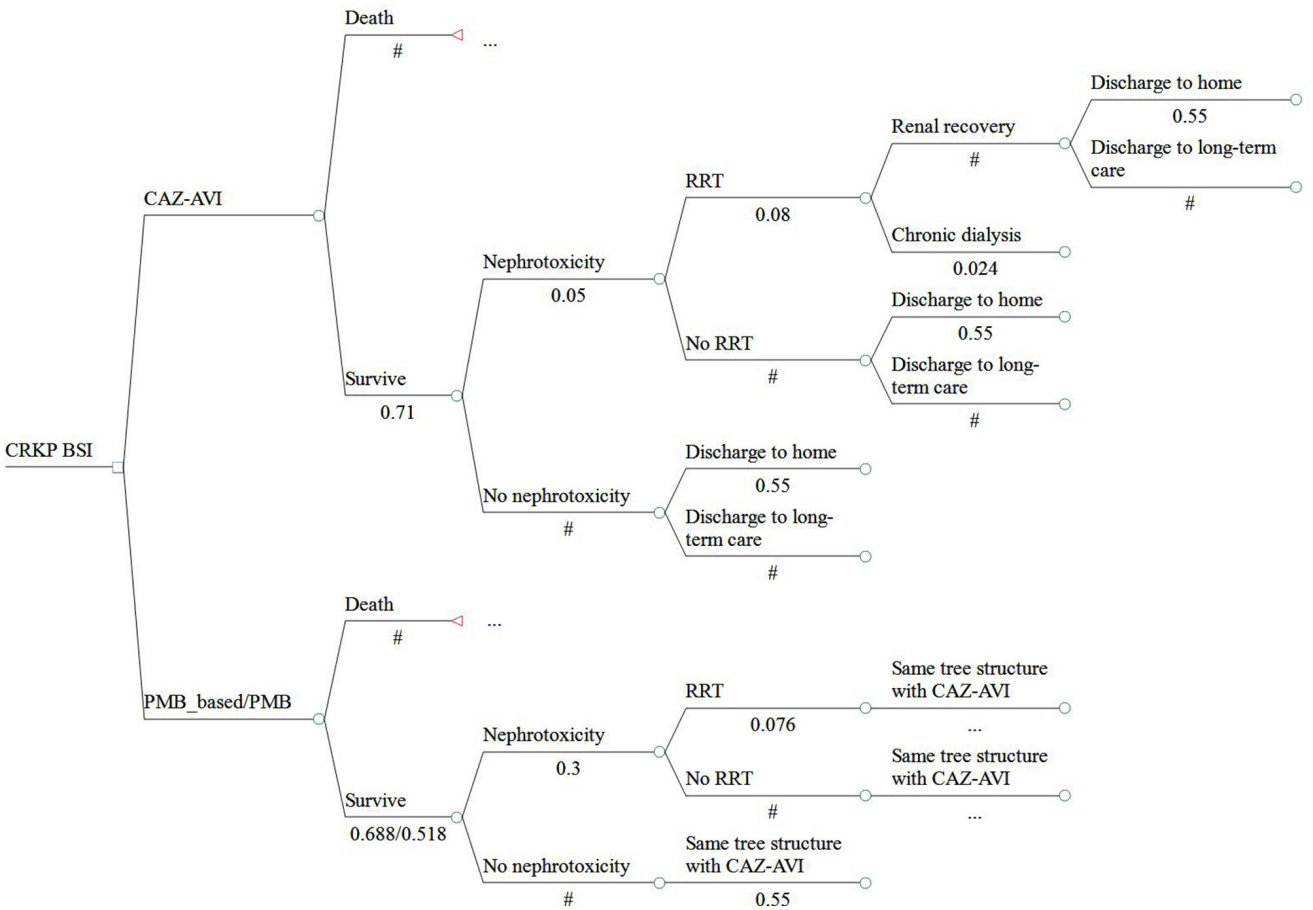
Structure of the model analysis. CRKP, carbapenem-resistant *Klebsiella pneumoniae*; BSI, bloodstream infection; RRT, renal replacement therapy; DC, discharge; LTC, long-term care.

**Figure 2:**
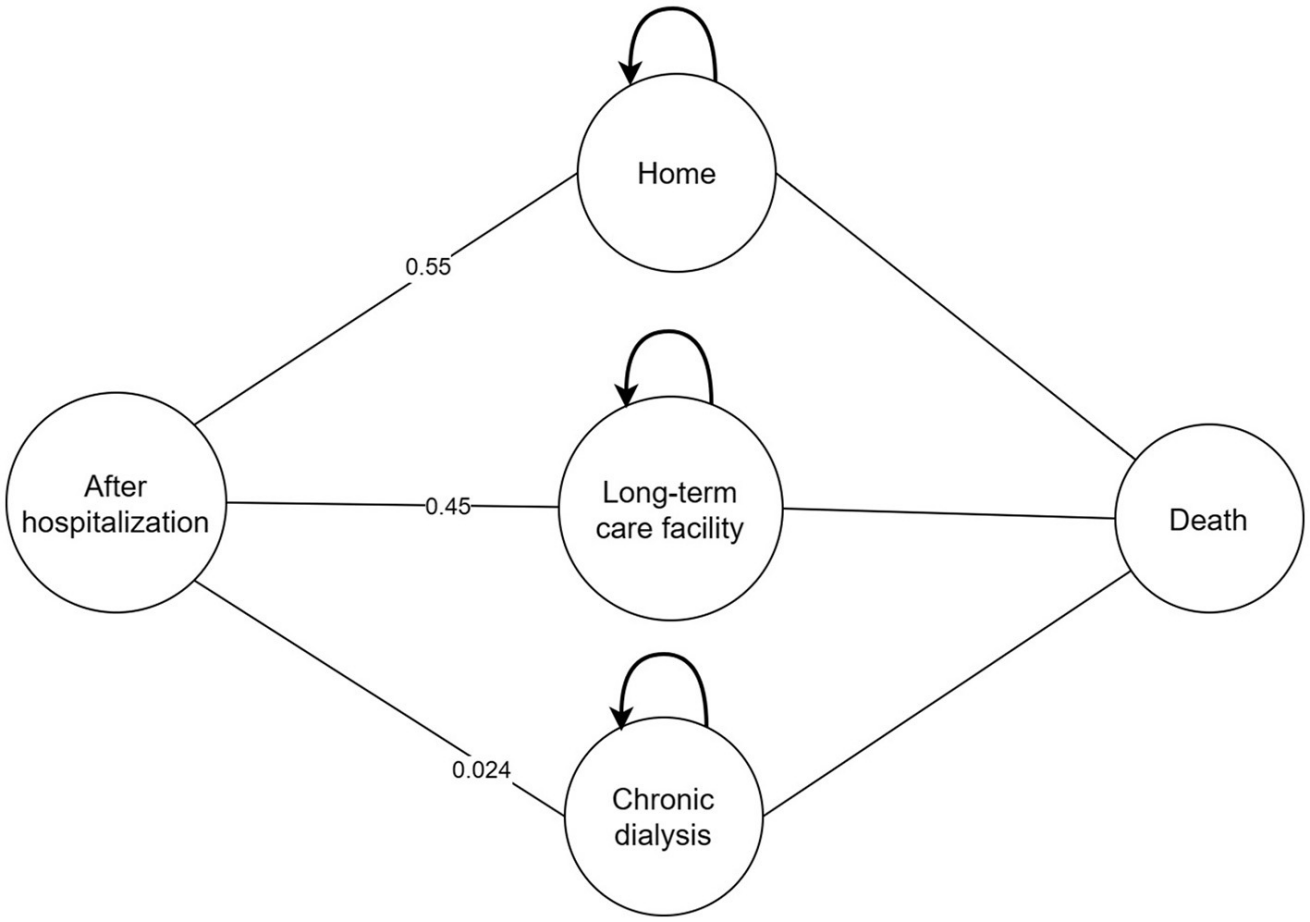
Markov model structure.

A cost-effectiveness analysis was performed based on a combined decision tree model and 5- year Markov model with a yearly cycle from the perspective of Chinese healthcare. We only calculated direct medical costs. We simulated a scenario wherein patients with a confirmed CRKP BSI were assigned to receive CAZ-AVI or PMB or PMB-based therapy. The total cohort represents 3,000 patients and 1,000 in each treatment strategy. In the decision tree model, total cost and quality adjusted life years (QALYs) were calculated for three regimens as definitive therapy in patients diagnosed with BSI caused by CRKP. Patients entered into the model had an equal probability of receiving any of therapy regimen. Patients can be treated successfully or can die due to BSI. Patients who are cured may develop nephrotoxicity which could be recoverable or require chronic dialysis. After hospitalization, patients may be discharged to home, discharged to a long-term care facility or chronic dialysis and entered into the 5-year Markov model linked to the decision tree model. Due to the lack of data, a 5-year Markov model was considered to estimate differences among therapy regimens in terms of costs and effectiveness and in line with other published economic studies (18, 20–22). Four states were presented in the Markov model including: 1. home; 2. long-term care facility; 3. chronic dialysis; 4. death. The models were presented in Figures 1, 2.

### Input parameters

Model inputs were extracted primarily from published literature and publicly available database. Clinical cure rate and nephrotoxicity among patients receiving CAZ-AVI, PMB, or PMB-based regimen were derived from systematic review and meta-analyses (15, 23). Treatment dose inputs were based on product labels. Among patients who developed nephrotoxicity, the probability of requiring renal replacement therapy (RRT) was estimated as the weighted average value by pooled multiple studies that reported the outcome for patients treated with CAZ-AVI, PMB or PMB-based regimens (Supplementary Table 1). The highest and lowest values retrieved from the studies were considered as the range included in the sensitivity analyses (Supplementary Table 1). The probability of a home discharge (55%) determined from a report of the China CRE network was assumed to be equivalent regardless of treatments (24). The risk (2.4%) of nephrotoxicity requiring chronic dialysis was derived from a meta-analysis compared colistin with PMB for the treatment of patients with multidrug-resistant gram-negative infections (25). We did not obtain the probability of chronic dialysis for patients receiving CAZ-AVI; thus, we assumed that the risk of chronic dialysis was equivalent to that PMB. The rates of a 5-year all-cause mortality in home or long-term care and a 5-year mortality on chronic dialysis were obtained from published economic studies (20, 21). A 14- day anti-infective duration was considered. The unit price of antibiotics was retrieved from the Yaozh database that collects successful biding prices of drugs in China (26). The daily costs of the antibiotics of interest are shown in Supplementary Table 2. As PMB-based regimen includes multiple antibiotics treatment (e.g., tigecycline, carbapenems, amikacin) for the treatment of CRKP BSI, the cost of PMB-based therapy was calculated as the weighted average according to a study published by Simon et al. (20). We retrieved the lowest prices on tigecycline, carbapenems, and amikacin. Other costs were retrieved from published literature (27–29). We assumed the costs of nephrotoxicity and long-term health care to be equivalent among three treatment strategies. Health utilities were obtained based on published literature (29–31). All costs were inflated to the 2021 value, according to the Chinese Health Consumer Price index (32) and converted into United States dollars ($) based on an exchange rate of $ 1 = ¥ 7.048. Utility values were 0.61, 0.84, and 0.64, respectively, for chronic dialysis, discharge to home, and discharge to long-term care. The key input parameters are summarized in Table 1.

**Table 1:** Model inputs for model analysis.

**Model input**	**Base-case value**	**Uncertainty range**	**Distribution**	**References**
**Cure**				
Ceftazidime-avibactam	0.71	0.63–0.85	Beta	(15)
Polymyxin B- based regimen	0.688	0.412–1.0	Beta	(23)
Polymyxin B	0.518	0.211–0.769	Beta	(23)
**Nephrotoxicity**				
Ceftazidime-avibactam	0.05	0–0.25	Beta	(15)
Polymyxin B- based regimen	0.30	0.24–0.37	Beta	(33)
Polymyxin B	0.30	0.24–0.37	Beta	(33)
Nephrotoxicity requiring RRT in hospital (polymyxin B)	0.076	0.034–0.159	Beta	Supplementary Table 3
Nephrotoxicity requiring RRT in hospital (ceftazidime-avibactam)	0.08	0–0.1	Beta	Supplementary Table 3
Nephrotoxicity requiring long-term RRT	0.024	0–0.05	Beta	(25)
Discharge to home	0.55	–	Beta	(24)
Duration of therapy (days)	14	–		
**All-cause mortality in renal recovery patients (home)**				
Year 1	0.356	0.15–0.55	Beta	(20)
Year 2-year 5	0.112	0.05–0.25	Beta	
**All-cause mortality in renal recovery patients (long-term care)**				
Year 1	0.479	0.2–0.6	Beta	(20)
Year 2-year 5	0.217	0.1–0.3	Beta	(20)
Death on chronic dialysis				(21)
Year 1	0.614	–	Beta	
Year 2	0.703	–	Beta	
Year 3	0.754	–	Beta	
Year 4	0.892	–	Beta	
Year 5	0.924	–	Beta	
**All-cause mortality without RRT patients**				(21)
Year 1	0.296			
Year 2	0.303			
Year 3	0.31			
Year 4	0.318			
Year 5	0.326			
**Cost (U.S. dollars)**				
Hemodialysis (annual)	30,143.2	2,7091.3–34,028.2	Gamma	(29)
Nephrotoxicity with RRT in hospital	11,720.7	5,246–23,346.2	Gamma	(27)
Nephrotoxicity without RRT in hospital	5,602.9	2,970–13,628.6	Gamma	(27)
Long-term care	2,434.1	±50%	Gamma	(28)
Polymyxin B- based regimen (daily)^*^	682.3	±50%	Gamma	
Ceftazidime-avibactam (daily)	594.2	±50%	Gamma	
Polymyxin B (daily)	653.6	±50%	Gamma	
**Heath utility**				
Chronic dialysis	0.61	0.59–0.63	Beta	(27)
Discharge to home	0.84	0.4–0.95	Beta	(30)
Discharge to long-term care	0.64	0.4–0.8	Beta	(31)

^*^The polymyxin B_based regimen included polymyxin B therapy (100%), tigecycline (61%), meropenem (51%), amikacin (23%), and gentamicin (14%) (20). RRT: renal replacement treatment.

### Outcomes

Total costs and QALYs were estimated for different treatment regimens. The treatment regimen was considered as highly cost-effective if an incremental cost-effectiveness ratio (ICER) was less than a given willingness-to-pay (WTP) of $ 11,600 per QALY, which was set to be a one-time Chinese gross domestic product (GDP) per capita in 2021, according to the Chinese guidelines (34). Annual discount rates of 5% was applied to all future costs and health benefits (34). All analyses were performed using the TreeAge Pro 2011 software.

### Sensitivity analyses

Deterministic and probabilistic sensitivity analyses in the model were conducted to examine the robustness of the model results owing to an uncertainty of input parameters (Table 1). The variations of deterministic sensitivity analysis included cure rates of antibiotics, costs of therapy (i.e., antibiotics and RRT), and probability of nephrotoxicity (Table 1). Results of the one-way deterministic sensitivity analyses (DSA) were shown as tornado diagrams. A probabilistic sensitivity analysis (PSA) was conducted by simultaneously executing 10,000 Monte Carlo simulations. The continuous variables (e.g., cost) were assumed to follow gamma distributions. The beta distribution was considered for binary variables (e.g., probability). The standard error of 10% of the average value was considered for all variables. Results of PSA were represented as cost-effectiveness acceptability curve.

## Results

### Base-case analysis

The results of base-case analysis are demonstrated in Table 2. During the 5- year time horizon, the CAZ-AVI strategy cost $ 23,261,700 with 1,240 QALYs. The PMB strategy cost $ 23,053,300 with 890 QALYs. The PMB-based strategy cost $25,480,000 with 1,180 QALYs. The ICER for CAZ-AVI was $ 591.7/QALY when compared to the PMB strategy, which suggested that CAZ-AVI was cost-effective at the threshold of $ 11,600 per QALY in the treatment of patients with CRKP BSI although it increased cost. For treated with CAZ-AVI, we observed a negative ICER of $ −36,730.9 per QALY gained. Negative ICER indicates that CAZ-AVI for the treatment of confirmed CRKP BSI, relative to PMB-based strategy, could be not only cost-effective but also cost-saving. Additionally, the ICER for PMB-based strategy was $ 8,369.97/QALY when compared with PMB alone, indicating that PMB-based strategy was cost-effective at the threshold of $ 11,600 per QALY.

**Table 2:** Base-case analysis results in 1,000 patients due to Carbapenem-Resistant *Klebsiella pneumoniae*.

**Treatment strategy**	**Cost ($)**	**No. of QALYs**	**Incremental cost ($)**	**Incremental QALYs**	**ICER ($/QALY)**
CAZ-AVI	23,261,700	1,240	–	–	–
PMB	23,053,300	890	208,400	350	591.7
PMB- based regimen	25,480,000	1,180	−2,218,300	60	−36,730.9 (Dominated)

CAZ-AVI: Ceftazidime-avibactam; PMB: Polymyxin B.

### Sensitivity analysis

**Figure 3:**
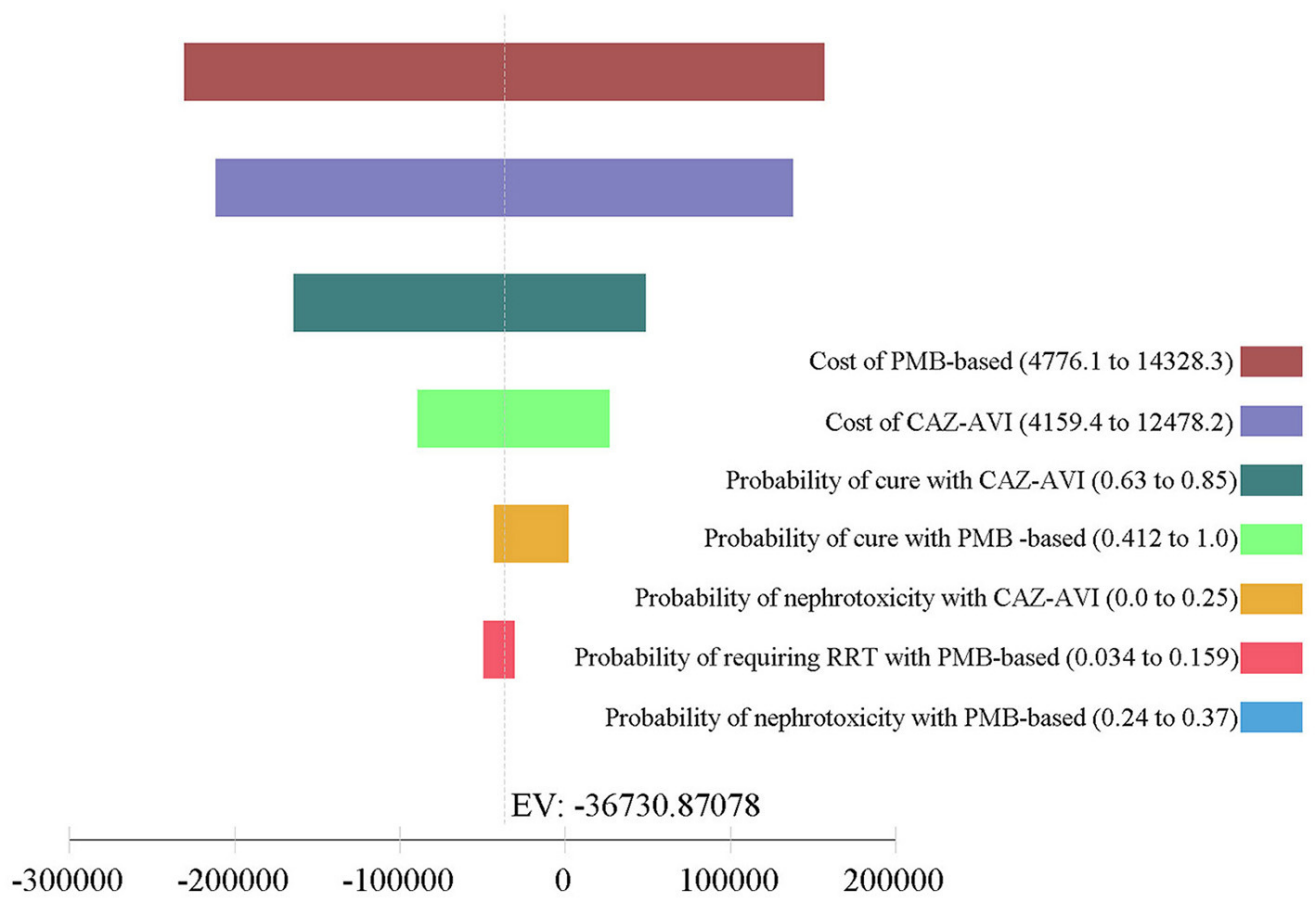
Tornado analysis depicting results of one-way sensitivity analysis of key variables for CAZ-AVI vs. PMB-based therapy. CAZ-AVI, Ceftazidime-avibactam; PMB, Polymyxin B.

**Figure 4:**
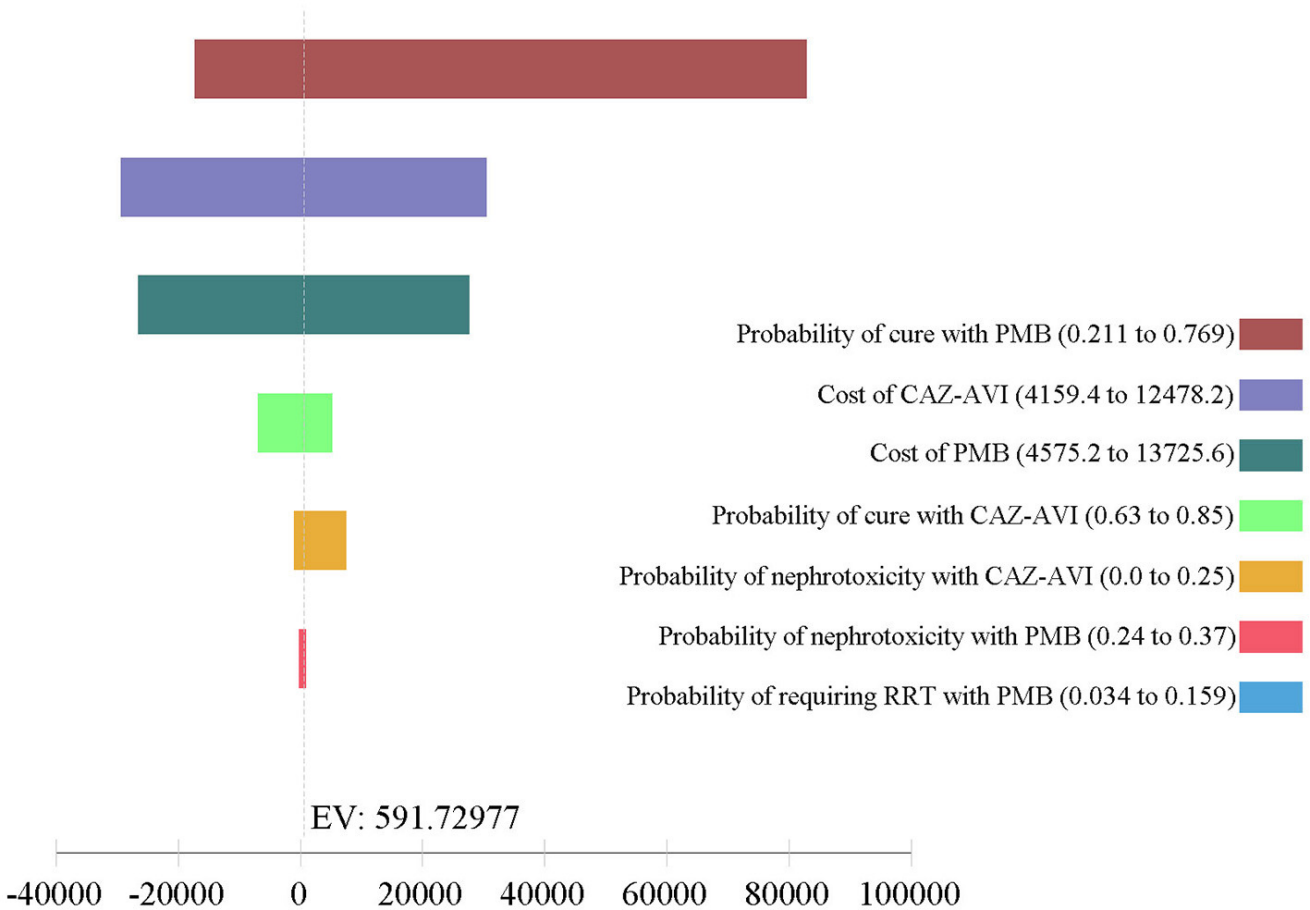
Tornado analysis depicting results of one-way sensitivity analysis of key variables for CAZ-AVI vs. PMB therapy. CAZ-AVI, Ceftazidime-avibactam; PMB, Polymyxin B; RRT, renal replacement therapy.

The results of one-way DSA are shown in Figures 3, 4. At the WTP threshold, the one-way DSA showed that the most influential parameters were the cost of PMB-based strategy, the cost of CAZ-AVI strategy, the probability of cure with CAZ-AVI, and the probability of cure with PMB -based when CAZ-AVI strategy was compared to PMB-based strategy for patients with CRKP BSI. The PMB-based strategy would be a more cost-effective option than CAZ-AVI, if the price had an approximately 13% reduction for PMB-based regimens based on one-way sensitivity analysis when the WTP threshold was set to $ 11,600. One-way DSA indicated that the cure rate of PMB, the cost of PMB strategy, and the cost of CAZ-AVI strategy had high impacts on the ICER of CAZ-AVI vs. PMB.

**Figure 5:**
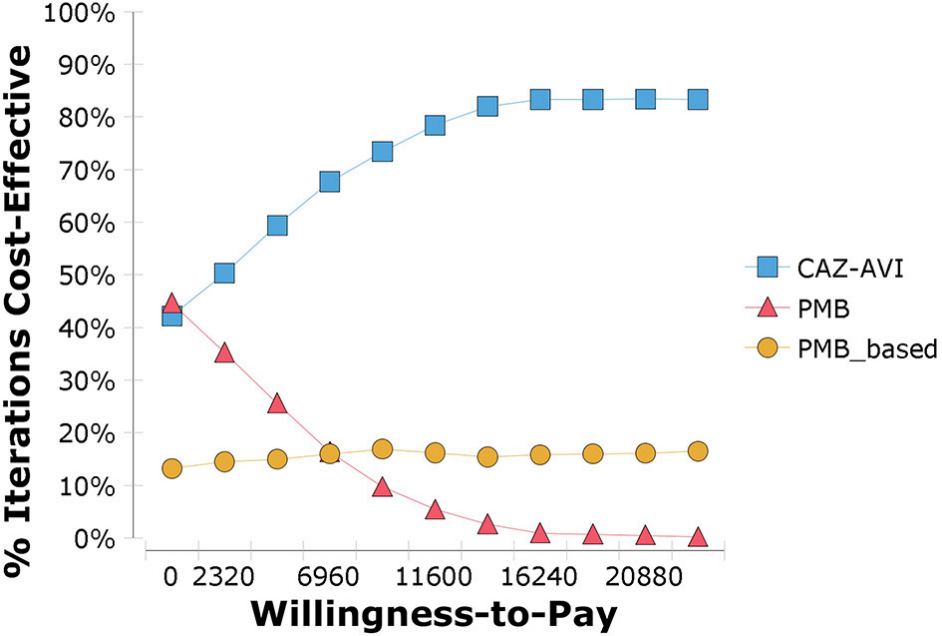
Cost-effectiveness acceptability curve showing the probability that ceftazidime-avibactam is cost-effective. CAZ-AVI, Ceftazidime-avibactam; PMB, Polymyxin B.

The results of PSA are in line with the base-case analysis, in that the probability that CAZ-AVI was cost-effective at the threshold of $11,600/QALY was 76.9% and at the threshold of three times the Chinese GDP per capita per QALY ($34,800/QALY) was 80.8%. The acceptability curve is shown in Figure 5. We also explored the probability of CAZ-AVI being cost-effective when compared with province-level WTP thresholds (one-time the province-level GDP per capita) (32). Compared with the comparators, the probability of CAZ-AVI being cost-effective at the province-level WTP thresholds ranged from 61.4% (Gansu) to 83.1% (Beijing). The results of PSA under province-level WTP thresholds are presented in Table 3.

**Table 3:** Results of PSA at the province-level WTP thresholds.

**No**.	**Province**	**GDP per capita in 2021 (¥)**	**WTP threshold ($) ^a^**	**Compared with PMB or PMB-based**	
				**ICER compared with the WTP threshold**	**Probability of cost-effectiveness (%)**
1	Beijing	183,980	26,343.4	Lower	83.1
2	Shanghai	173,630	24,861.5	Lower	81.9
3	Jiangsu	137,039	19,622.1	Lower	81.7
4	Fujian	116,939	16,744.1	Lower	81.4
5	Tianjin	113,732	16,284.9	Lower	82.7
6	Zhejiang	113,032	16,184.7	Lower	83.4
7	Guangdong	98,285	14,073.1	Lower	81.4
8	Chongqing	86,879	12,439.9	Lower	81.6
9	Hubei	86,416	12,373.6	Lower	78.7
10	Neimenggu	85,422	12,231.3	Lower	79.9
11	Shandong	81,727	11,702.2	Lower	78.1
12	Shanxi	75,360	10,790.5	Lower	75.4
13	Anhui	70,321	10,069.0	Lower	77.2
14	Hunan	69,440	9,942.9	Lower	73.8
15	Jiangxi	65,560	9,387.3	Lower	74.8
16	Liaoning	65,026	9,310.8	Lower	74.7
17	Shanxi	64,821	9,281.5	Lower	74.6
18	Sichuan	64,326	9,210.6	Lower	74.5
19	Hainan	63,707	9,122	Lower	71.5
20	Ningxia	62,549	8,956.2	Lower	73.5
21	Xinjiang	61,725	8,838.2	Lower	71.2
22	Henan	59,410	8,506.7	Lower	70.5
23	Yunnan	57,686	8,259.9	Lower	70.7
24	Xizang	56,831	8,137.4	Lower	69.7
25	Qinghai	56,398	8,075.4	Lower	71.3
26	Jilin	55,450	7,939.7	Lower	69.6
27	Hebei	54,172	7,756.7	Lower	69.7
28	Guizhou	50,808	7,275.0	Lower	67.8
29	Guangxi	49,206	7,045.6	Lower	66.8
30	Heilongjiang	47,266	6,767.9	Lower	65
31	Gansu	41,046	5,877.2	Lower	61.4

^a^The WTP threshold was set at one time the province-level GDP per capita in 2021.

GDP, gross domestic product; ICER, incremental cost-effectiveness ratio; WTP, willingness-to-pay; PMB, polymyxin B.

## Discussion

To our knowledge, this is the first study to assess the cost-effectiveness of CAV-AVI for the treatment of BSI caused by CRKP in China. Given the excess mortality and significant economic burden associated with CRKP, assessing CAZ-AVI's value is critical to understanding its potential impact in treating CRKP with limited treatment options (6, 7). Several pharmacoeconomic studies of CAZ-AVI have been conducted in the USA, Netherlands, and Italy in infectious diseases (17, 18, 20). These published literatures have consistently indicated that CAZ-AVI was cost-effective at the WTP thresholds for the treatment of various infective diseases such as cIAIs, cUTIs, CRE bacteremia, and pneumonia. The key strength of the present study is that it is the first to focus on the treatment of patients with BSI caused by CRKP that has led to high mortality and heavy economic burden. Another strength is comparing the cost-effectiveness of an existing drug (PMB) and a novel BL/BLI (ceftazidime-avibactam) as definitive treatments for CRKP BSI, which are both expensive in China. Meanwhile, differences in economic development among regions in China were well considered in this model analysis.

The base-case results of the present study showed that ceftazidime-avibactam is highly cost-effective when compared with PMB monotherapy or combination therapy in patients with CRKP BSI, from the perspective of Chinese healthcare, as the ICERs saved by CAZ-AVI were well below the WTP threshold. The study findings were consistent with those of the recent cost-effectiveness analysis in the United States, wherein compared with colistin, CAZ-AVI was cost-effective for the treatment of CRE bloodstream infection (20). Although the International Consensus Guidelines for the Optimal Use of Polymyxins (12) did not recommend PMB alone for the treatment of invasive CRE infections given that monotherapy was associated with higher mortality, we considered that a cost-effectiveness study of PMB monotherapy vs. PMB combination therapy may be crucial when CAZ-AVI may not be available in some clinical settings. The present study found that PMB-based strategy cost an additional $ 2,426.7 and gained 0.29 QALY per infection cases, and the ICER was $ 8,367.9/QALY, indicating that PMB-based strategy was cost-effective at a threshold of the WTP compared with PMB monotherapy. The results of probabilistic sensitivity analysis supported CAZ-AVI to be cost-effective in over 76% of 10 000 Monte Carlo simulations at a WTP of one-time GDP in China. Moreover, the results remain robust in the lowest income province in China. In 10,000 Monte Carlo simulations at a WTP of one-time GDP in Gansu, over 61% simulations showed incremental QALYs gained by CAZ-AVI. One-way sensitivity analysis indicated that the cost of PMB-based regimens had the most impact on the ICER of CAZ-AVI vs. PMB-based regimens. At the WTP threshold, the daily cost of PMB-based regimens (vs. CAZ-AVI) at less than about $ 593.6, would make it cost-effective. There was an average price reduction of 52% for bid-winning drugs such as fluconazole and itraconazole due to the Chinese National Centralized Drug Procurement (NCDP) policy in 2019 (35). Therefore, a significant price reduction of PMB could be anticipated with the implementation of the NCDP. An updated cost-effectiveness analysis is warranted in the future. The clinical cure of PMB monotherapy also had the largest impact on ICER of CAZ-AVI vs. PMB, which was unsurprising in that lower clinical cure reduced the proportion of patients who entered the RRT treatment and 5-year model, thereby influencing the overall treatment cost. The clinical cures of PMB monotherapy and PMB combination therapy were obtained from a meta-analysis favoring polymyxins combination therapy for *K. pneumoniae* bacteremia (23). Although the recommendation of polymyxins combination therapy for CRE infections is a controversial topic given the limited and low-quality evidence (12), the results of this meta-analysis based on synthesizing data from observational studies would improve confidence in our study given as a randomized controlled trial is unlikely to be conducted to address the controversial topic.

A multicenter, retrospective research from the China CRE network demonstrated an overall CRE infection incidence rate of 4.0 per 10,000 discharges. Most of the infection was caused by KP primarily producing KPC-2 enzyme (24). Zhu et al. found that KPC-producing *K. pneumoniae* BSIs were associated with higher medical costs, and the median burden for single patient was approximately $59,366.2, which is significantly higher than the incomes of the average person in China (6). The reported CRE incidence was 2.93 per 100,000 population in the United States (36). The Centers for Disease Control and Prevention estimated about 9,000 CRE infections annually in the United States, and approximately 46% of these are either BSI or pneumonia (20). If all patients with BSI were treated using CAZ-AVI as first-line therapy, instead of PMB-based strategy, roughly saved $ 9.18 million could be saved and gained 248.4 QALYs gained in China. Thus, it is worth increasing the use of CAZ-AVI based on China's accepted willingness to pay standards.

Our model analysis has some limitations that should be noted. First, we calculated the daily dose of PMB according to the label's recommendation, which is lower than the recommendation of International Consensus Guidelines (12) and clinical literature (37). A loading dose of 2.0–2.5 mg/kg, followed by a maintenance dose of 1.25–1.5 mg/kg every 12 h is recommended by those studies, which may result in higher daily cost of PMB. If the recommended dose of PMB had been included in our model analysis, this study may have underestimated the cost-effectiveness of ceftazidime-avibactam considering the cost of PMB-based strategy had a highly impact on the ICER. However, a daily dose adopted from the insert package is safer, because polymyxin-associated nephrotoxicity is associated with the magnitude of polymyxin exposure (12). Second, nephrotoxicity-associated costs (i.e., costs associated with prolonged hospitalization due to nephrotoxicity) other than the cost of RRT were ignored in our model analysis. Given that the risk of nephrotoxicity receiving PMB is much higher than that of receiving CAZ-AVI, our analysis may further underestimate the cost-effectiveness of CAZ-AVI. Third, *K. pneumoniae* was assumed to be susceptible to CAZ-AVI due to the detection rate of KPC carbapenemase was high in China (10, 38). Nevertheless, *bla*NDM was the most common resistance gene detectable among *K. pneumoniae* in some regions such as Shanxi (38). In these regions, PMB-based regimens may be the preferred option for the treatment of CRKP BSI. Fourth, since observational studies with small simple sizes served as our clinical data source for cure rates and nephrotoxicity, these clinical data may not be robust given that observational studies are inevitably proven to confounders and bias. However, the efficacy and safety of CAZ-AVI and PMB for the treatment of patients with CRKP may be hard to evaluate through randomized controlled trials. Thus, the clinical data based on multiple observational studies is acceptable in the present scenario. Fifth, our model did not take the relapse rates and rehospitalizations into account. Chen et al. conducted a meta-analysis and found the relapse rate was similar between the CAZ-AVI group and the comparator group. Meanwhile, the impact of CAZ-AVI compared with PMB-based regimen on rehospitalization rates lacked data (20). Sixth, because of unavailability of data on the long-term mortality and the health utility of patients with CRKP BSI, the model utilized published data of other populations. Studies specific to CRKP BSI that determine the long-term mortality and health utility of patients are necessary. Seventh, the findings from our study may not be suitable for other counties due to significant variations in healthcare resource and epidemiology of *K. pneumoniae* resistance across different countries. Lastly, our data for antibiotic costs was based on average bidding prices reported from the Yaozh database, which may not reflect the true costs of all Chinese provinces.

## Conclusion

Our study results indicate that CAZ-AVI is cost-effective as a definitive treatment among patients with CRKP BSI, ensuring that patients had better health outcomes overall. Thus, CAZ-AVI should be considered as an alternative to the PMB-based strategy, because CAZ-AVI not only produces better health outcomes but also helps extend PMB's lifecycle, serving as the last-line antibiotic for treating multidrug-resistant, gram-negative bacterial infections.

## Data availability statement

The original contributions presented in the study are included in the article/Supplementary material, further inquiries can be directed to the corresponding author.

## Ethics statement

All the data included in this analysis were derived from published literature and public data. No patient- identifiable data were applied or used. Therefore, institutional review board approval was not required.

## Author contributions

WK and XY contributed to the design of this study. YS and SL collected the data. WK prepared the manuscript. BS and KY helped to revise the manuscript. All authors approved the final version of this study.

## References

[1] Murray CJ Ikuta KS Sharara F Swetschinski L Aguilar GR Gray A. Global burden of bacterial antimicrobial resistance in 2019: a systematic analysis. Lancet. (2022) 399:629–55. 10.1016/S0140-6736(21)02724-0 35065702 PMC8841637

[2] Munoz-Price LS Poirel L Bonomo RA Schwaber MJ Daikos GL Cormican M. Clinical epidemiology of the global expansion of klebsiella pneumoniae carbapenemases. Lancet Infect Dis. (2013) 13:785–96. 10.1016/S1473-3099(13)70190-7 23969216 PMC4673667

[3] Pouch SM Satlin MJ. Carbapenem-resistant enterobacteriaceae in special populations: solid organ transplant recipients, stem cell transplant recipients, and patients with hematologic malignancies. Virulence. (2017) 8:391–402. 10.1080/21505594.2016.1213472 27470662 PMC5477691

[4] Satlin MJ Jenkins SG Walsh TJ. The global challenge of carbapenem-resistant enterobacteriaceae in transplant recipients and patients with hematologic malignancies. Clin Infect Dis. (2014) 58:1274–83. 10.1093/cid/ciu052 24463280 PMC4038783

[5] Tumbarello M Trecarichi EM De Rosa FG Giannella M Giacobbe DR Bassetti M. Infections caused by Kpc-producing klebsiella pneumoniae: differences in therapy and mortality in a multicentre study. J Antimicrob Chemother. (2015) 70:2133–43. 10.1093/jac/dkv200 25900159

[6] Zhu Y Xiao T Wang Y Yang K Zhou Y Luo Q. Socioeconomic burden of bloodstream infections caused by carbapenem-resistant enterobacteriaceae. Infect Drug Resist. (2021) 14:5385–93. 10.2147/IDR.S341664 34938086 PMC8685763

[7] Xu L Sun X Ma X. Systematic review and meta-analysis of mortality of patients infected with carbapenem-resistant *Klebsiella Pneumoniae*. *Ann Clin Microbiol Antimicrob*. (2017) 16:18. 10.1186/s12941-017-0191-3 28356109 PMC5371217

[8] Tacconelli E Carrara E Savoldi A Harbarth S Mendelson M Monnet DL. Discovery, research, and development of new antibiotics: the who priority list of antibiotic-resistant bacteria and tuberculosis. Lancet Infect Dis. (2018) 18:318–27. 10.1016/S1473-3099(17)30753-3 29276051

[9] Yan M Zheng B Li Y Lv Y. Antimicrobial susceptibility trends among gram-negative bacilli causing bloodstream infections: results from the China antimicrobial resistance surveillance trial (Carst) program, 2011–2020. Infect Drug Resist. (2022) 15:2325–37. 10.2147/IDR.S358788 35517902 PMC9064452

[10] Han R Shi Q Wu S Yin D Peng M Dong D. Dissemination of carbapenemases (Kpc, Ndm, Oxa-48, Imp, and Vim) among carbapenem-resistant enterobacteriaceae isolated from adult and children patients in China. Front Cell Infect Microbiol. (2020) 10:314. 10.3389/fcimb.2020.00314 32719751 PMC7347961

[11] Guan X He L Hu B Hu J Huang X Lai G. Laboratory diagnosis, clinical management and infection control of the infections caused by extensively drug-resistant gram-negative bacilli: a chinese consensus statement. Clin Microbiol Infect. (2016) 22:S15–25. 10.1016/j.cmi.2015.11.004 26627340

[12] Tsuji BT Pogue JM Zavascki AP Paul M Daikos GL Forrest A. International Consensus Guidelines for the Optimal Use of the Polymyxins: Endorsed by the American College of Clinical Pharmacy (Accp), European Society of Clinical Microbiology and Infectious Diseases (Escmid), Infectious Diseases Society of America (Idsa), International Society for Anti-Infective Pharmacology (Isap), Society of Critical Care Medicine (Sccm), and Society of Infectious Diseases Pharmacists (Sidp). Pharmacotherapy. (2019) 39:10–39. 10.1002/phar.2209 30710469 PMC7437259

[13] Zavascki AP Nation RL. Nephrotoxicity of polymyxins: is there any difference between colistimethate and polymyxin B? Antimicrobial Agents Chemotherapy. (2017) 61:e02319–16. 10.1128/AAC.02319-16 27993859 PMC5328560

[14] van Duin D Bonomo RA. Ceftazidime/avibactam and Ceftolozane/Tazobactam: Second-Generation B-Lactam/B-Lactamase Inhibitor Combinations. Clin Infect Dis. (2016) 63:234–41. 10.1093/cid/ciw243 27098166 PMC4928383

[15] Chen Y Huang HB Peng JM Weng L Du B. Efficacy and safety of ceftazidime-avibactam for the treatment of carbapenem-resistant enterobacterales bloodstream infection: a systematic review and meta-analysis. Microbiol Spectrum. (2022) 10:e0260321. 10.1128/spectrum.02603-21 35377233 PMC9045088

[16] WHO. 21st Who Model List of Essential Medicines. (2019). Available online at: https://www.who.int/publications/i/item/WHO-MHP-HPS-EML-2021.02 (accessed November 10, 2022).

[17] Kongnakorn T Eckmann C Bassetti M Tichy E Di Virgilio R Baillon-Plot N. Cost-Effectiveness Analysis Comparing Ceftazidime/Avibactam (Caz-Avi) as Empirical Treatment Comparing to Ceftolozane/Tazobactam and to Meropenem for Complicated Intra-Abdominal Infection (Ciai). Antimicrob Resist Infect Control. (2019) 8:204. 10.1186/s13756-019-0652-x 31890160 PMC6925481

[18] Kongnakorn T Wagenlehner F Falcone M Tichy E Di Virgilio R Baillon-Plot N. Cost-effectiveness analysis of ceftazidime/avibactam compared to imipenem as empirical treatment for complicated urinary tract infections. Int J Antimicrob Agents. (2019) 54:633–41. 10.1016/j.ijantimicag.2019.06.008 31202921

[19] Han R Teng M Zhang Y Zhang T Wang T Chen J. Choosing optimal antibiotics for the treatment of patients infected with enterobacteriaceae: a network meta-analysis and cost-effectiveness analysis. Front Pharmacol. (2021) 12:656790. 10.3389/fphar.2021.656790 34220501 PMC8245689

[20] Simon MS Sfeir MM Calfee DP Satlin MJ. Cost-effectiveness of ceftazidime-avibactam for treatment of carbapenem-resistant *Enterobacteriaceae Bacteremia* and Pneumonia. Antimicrobial Agents Chemotherapy. (2019) 63:e00897–19. 10.1128/AAC.00897-19 31548187 PMC6879229

[21] Mennini FS Gori M Vlachaki I Fiorentino F Malfa P Urbinati D. Cost-effectiveness analysis of vaborem in carbapenem-resistant enterobacterales (Cre) -*klebsiella* pneumoniae infections in Italy. Health Econ Rev. (2021) 11:42. 10.1186/s13561-021-00341-z 34716794 PMC8557067

[22] Nguyen CP Dan Do TN Bruggemann R Ten Oever J Kolwijck E Adang EMM. Clinical cure rate and cost-effectiveness of carbapenem-sparing beta-lactams vs. meropenem for gram-negative infections: a systematic review, meta-analysis, and cost-effectiveness analysis. Int J Antimicrob Agents. (2019) 54:790–7. 10.1016/j.ijantimicag.2019.07.003 31284041

[23] Zusman O Altunin S Koppel F Dishon Benattar Y Gedik H Paul M. Polymyxin monotherapy or in combination against carbapenem-resistant bacteria: systematic review and meta-analysis. J Antimicrob Chemother. (2017) 72:29–39. 10.1093/jac/dkw377 27624572

[24] Zhang Y Wang Q Yin Y Chen H Jin L Gu B. Epidemiology of carbapenem-resistant enterobacteriaceae infections: report from the China cre network. Antimicrobial Agents Chemotherapy. (2018) 62:e01882–17. 10.1128/AAC.01882-17 29203488 PMC5786810

[25] Vardakas KZ Falagas ME. Colistin versus polymyxin b for the treatment of patients with multidrug-resistant gram-negative infections: a systematic review and meta-analysis. Int J Antimicrob Agents. (2017) 49:233–8. 10.1016/j.ijantimicag.2016.07.023 27686609

[26] Yaozh. Yaozh Database. (2022). Available online at: https://db.yaozh.com/ (accessed October 21, 2022).

[27] Wang F Hong D Wang Y Feng Y Wang L Yang L. Renal replacement therapy in acute kidney injury from a chinese cross-sectional study: patient, clinical, socioeconomic and health service predictors of treatment. BMC Nephrol. (2017) 18:152. 10.1186/s12882-017-0567-9 28472927 PMC5418849

[28] Xu X Chen LJS. Projection of Long-Term Care Costs in China, 2020–2050, Based on the Bayesian Quantile Regression Method. (2019) 11:3530. 10.3390/su1113353036691990

[29] Yang F Liao M Wang P Liu Y. Cost-effectiveness analysis of renal replacement therapy strategies in Guangzhou City, Southern China. BMJ Open. (2021) 11:e039653. 10.1136/bmjopen-2020-039653 33550227 PMC7925861

[30] Bartsch SM McKinnell JA Mueller LE Miller LG Gohil SK Huang SS. Potential economic burden of carbapenem-resistant enterobacteriaceae (Cre) in the United States. Clin Microbiol Infect. (2017) 23:48 e9–e16. 10.1016/j.cmi.2016.09.003 27642178 PMC5547745

[31] MacNeil Vroomen JL Boorsma M Bosmans JE Frijters DH Nijpels G van Hout HP. Is it time for a change? A cost-effectiveness analysis comparing a multidisciplinary integrated care model for residential homes to usual care. PLoS ONE. (2012) 7:e37444. 10.1371/journal.pone.0037444 22655047 PMC3360056

[32] National Bureau of Statistic. (2021). Available online at: http://www.stats.gov.cn/tjsj/tjgb/ndtjgb/ (accessed October 21, 2022).

[33] Oliota AF Penteado ST Tonin FS Fernandez-Llimos F Sanches AC. Nephrotoxicity prevalence in patients treated with polymyxins: a systematic review with meta-analysis of observational studies. Diagn Microbiol Infect Dis. (2019) 94:41–9. 10.1016/j.diagmicrobio.2018.11.008 30635223

[34] Liu G. China Guidelines for Pharmacoeconomic Evaluations. Beijing: China Market Press (2020).

[35] Wang N Yang Y Xu L Mao Z Cui D. Influence of Chinese national centralized drug procurement on the price of policy-related drugs: an interrupted time series analysis. BMC Public Health. (2021) 21:1883. 10.1186/s12889-021-11882-7 34663282 PMC8524972

[36] Guh AY Bulens SN Mu Y Jacob JT Reno J Scott J. Epidemiology of carbapenem-resistant enterobacteriaceae in 7 us communities, 2012-2013. JAMA. (2015) 314:1479–87. 10.1001/jama.2015.12480 26436831 PMC6492240

[37] Wu X Huang C Wang H Ji J Ying C Xiao Y. Optimal empiric polymyxin B treatment of patients infected with gram-negative organisms detected using a blood antimicrobial surveillance network in China. Drug Des Devel Ther. (2021) 15:2593–603. 10.2147/DDDT.S313714 34168431 PMC8216662

[38] Zhang R Liu L Zhou H Chan EW Li J Fang Y. Nationwide surveillance of clinical carbapenem-resistant enterobacteriaceae (Cre) strains in China. EBioMed. (2017) 19:98–106. 10.1016/j.ebiom.2017.04.032 28479289 PMC5440625

