# Supplementary material for: Cost-effectiveness analysis of ceftazidime-avibactam as definitive treatment for treatment of carbapenem-resistant Klebsiella pneumoniae bloodstream infection
Source: Front Public Health. 2023 Feb 28;11:1118307. doi: 10.3389/fpubh.2023.1118307 (PMC10011158; doi:10.3389/fpubh.2023.1118307)
Supplement: Supplementary file 1 [file Data_Sheet_1.pdf]

Table S1. Studies used to estimate risk of requiring renal replacement therapy (RRT) among patients with nephrotoxicity on polymyxin B or ceftazidime-avibactam.

Author	Agent	Number of patients with AKI	Number of patients requiring RRT	%RRT
Kubin [1]	Polymyxin B	44	7	15.9%
Dubrovskaya [2]	Polymyxin B	88	3	3.4%
**Overall**	Polymyxin B	132	10	7.6% (3.4%-15.9%)
Shields [3]	Ceftazidime-avibactam	2	0	0%
Ackley [4]	Ceftazidime-avibactam	10	1	10%
**Overall**	Ceftazidime-avibactam	12	1	0.083% (0%-10%)

AKI: acute kidney injury

Table S2. Daily cost of the antibiotics of interest

**Medicine**	**Dosage and administration**	**Forms and strengths**	**Price per package ($)**	**Daily cost ($)**
Ceftazidime-avibactam	2.5 g every 8 h by intravenous	Powder, 2.5 g	198.06	594.2
Polymyxin B	0.5 million IU every 12 h by intravenous	Powder, 0.5 million IU	326.8	653.6
Tigecycline	Loading dose 100mg, following 50mg every 12 h by intravenous	Powder, 50mg	4.97	19.86
Meropenem	2g every 8 h by intravenous	Powder, 0.5g	1.52	18.22
Amikacin	0.4g qd by intravenous	Powder, 0.2g	10.78	21.56
Gentamicin	80 mg every 8 h by intravenous	Powder, 80mg	5.62	16.86

References

[1] Kubin CJ, Ellman TM, Phadke V, Haynes LJ, Calfee DP, Yin MT. Incidence and predictors of acute kidney injury associated with intravenous polymyxin B therapy. J Infect. 2012;65(1):80-7.

[2] Dubrovskaya Y, Prasad N, Lee Y, Esaian D, Figueroa DA, Tam VH. Risk factors for nephrotoxicity onset associated with polymyxin B therapy. J Antimicrob Chemother. 2015;70(6):1903-7.

[3] Shields RK, Nguyen MH, Chen L, Press EG, Potoski BA, Marini RV, Doi Y, Kreiswirth BN, Clancy CJ. Ceftazidime-Avibactam Is Superior to Other Treatment Regimens against Carbapenem-Resistant Klebsiella pneumoniae Bacteremia. Antimicrob Agents Chemother. 2017;61(8): e00883-17.

[4] Ackley R, Roshdy D, Meredith J, Minor S, Anderson WE, Capraro GA, Polk C. Meropenem-Vaborbactam versus Ceftazidime-Avibactam for Treatment of Carbapenem-Resistant Enterobacteriaceae Infections. Antimicrob Agents Chemother. 2020;64(5): e02313-19.
